# 4-Acetyl­pyridinium perchlorate

**DOI:** 10.1107/S1600536809024805

**Published:** 2009-07-08

**Authors:** Xue-qun Fu

**Affiliations:** a Ordered Matter Science Research Center, Southeast University, Nanjing 210096, People’s Republic of China

## Abstract

In the crystal of the title mol­ecular salt, C_7_H_8_NO^+^·ClO_4_^−^, the ions are linked by N—H⋯O hydrogen bonds, resulting in chains propagating in [010]. The packing is reinforced by C—H⋯O inter­actions.

## Related literature

For the synthesis, see: Piner (1934 ▶).
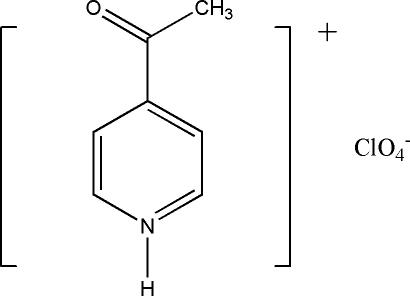


## Experimental

### 

#### Crystal data

C_7_H_8_NO^+^·ClO_4_^−^
*M* *_r_* = 221.59
Monoclinic, 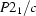

*a* = 5.4657 (11) Å
*b* = 12.621 (3) Å
*c* = 13.490 (3) Å
β = 97.88 (3)°
*V* = 921.8 (4) Å^3^
*Z* = 4
Mo *K*α radiation
μ = 0.41 mm^−1^
*T* = 298 K
0.20 × 0.20 × 0.20 mm

#### Data collection

Rigaku SCXmini diffractometer
Absorption correction: multi-scan (*CrystalClear*; Rigaku, 2005 ▶) *T*_min_ = 0.921, *T*_max_ = 0.921
9446 measured reflections
2108 independent reflections
1619 reflections with *I* > 2σ(*I*)
*R*_int_ = 0.049

#### Refinement

*R*[*F* ^2^ > 2σ(*F* ^2^)] = 0.062
*wR*(*F* ^2^) = 0.167
*S* = 1.06
2108 reflections
127 parameters
7 restraints
H-atom parameters constrained
Δρ_max_ = 0.65 e Å^−3^
Δρ_min_ = −0.90 e Å^−3^

### 

Data collection: *CrystalClear* (Rigaku, 2005 ▶); cell refinement: *CrystalClear*; data reduction: *CrystalClear*; program(s) used to solve structure: *SHELXS97* (Sheldrick, 2008 ▶); program(s) used to refine structure: *SHELXL97* (Sheldrick, 2008 ▶); molecular graphics: *SHELXTL* (Sheldrick, 2008 ▶); software used to prepare material for publication: *SHELXTL*.

## Supplementary Material

Crystal structure: contains datablocks I, global. DOI: 10.1107/S1600536809024805/hb5018sup1.cif

Structure factors: contains datablocks I. DOI: 10.1107/S1600536809024805/hb5018Isup2.hkl

Additional supplementary materials:  crystallographic information; 3D view; checkCIF report

## Figures and Tables

**Table 1:** Hydrogen-bond geometry (Å, °)

*D*—H⋯*A*	*D*—H	H⋯*A*	*D*⋯*A*	*D*—H⋯*A*
N1—H1*A*⋯O1^i^	0.86	2.14	2.896 (5)	146
C1—H1*B*⋯O5^ii^	0.93	2.49	2.963 (5)	112
C2—H2*A*⋯O3^iii^	0.93	2.59	3.435 (6)	151
C5—H5*A*⋯O4^i^	0.93	2.46	3.332 (6)	156
C7—H7*B*⋯O3^iii^	0.96	2.58	3.488 (6)	158

Symmetry codes: (i) 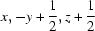; (ii) 
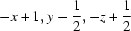
; (iii) 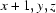.

**Fig. 1.**
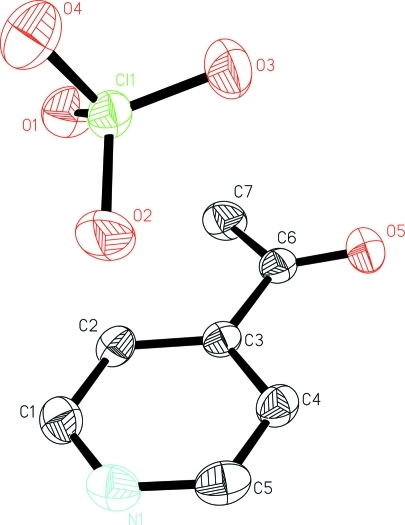
The molecular structure of (I) with displacement ellipsoids drawn at the 30% probability level (all H atoms have been omitted for clarity).

**Fig. 2.**
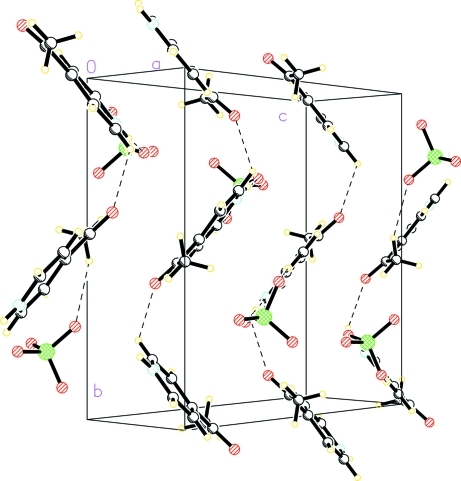
A view of the packing of (I) showing chains along the b axis. Dashed lines indicate hydrogen bonds.

## supplementary crystallographic information

### Comment

The asymmetric unit of the title compound contains a 4-Acetylpyridinium cation and a perchlorate anion (Fig 1). The bond length of O5—C6 and C6—C7 are 1.202 (5)Å and 1.492 (6)Å respectively, and the average bond length of Cl—O is 1.428 (3) Å. The N—H···O and C—H···O hydrogen bonding (Table 1) (N1—H···O1 2.896 (5) Å, C1—H···O5 2.963 (5) Å) make great contribution to the stability of the crystal structure and link the molecules to chains along the *b* axis (Fig 2).

### Experimental

4-Acetylpyridine was obtained according to the method described by Piner (1934) and colourless prisms of (I) were recrystallised from ethanol.

### Refinement

The positional parameters of all the H atoms were calculated geometrically and refined as riding with *U*_iso_(H) = 1.2*U*_eq_(carrier).

### Crystal data

**Table:**

C_7_H_8_NO^+^·ClO_4_^−^	*F*(000) = 456
*M**_r_* = 221.59	*D*_x_ = 1.597 Mg m^−^^3^
Monoclinic, *P*2_1_/*c*	Mo *K*α radiation, λ = 0.71073 Å
Hall symbol: -P 2ybc	Cell parameters from 4087 reflections
*a* = 5.4657 (11) Å	θ = 3.1–27.6°
*b* = 12.621 (3) Å	µ = 0.41 mm^−^^1^
*c* = 13.490 (3) Å	*T* = 298 K
β = 97.88 (3)°	Prism, colourless
*V* = 921.8 (4) Å^3^	0.20 × 0.20 × 0.20 mm
*Z* = 4	

### Data collection

**Table:**

Rigaku SCXmini diffractometer	2108 independent reflections
Radiation source: fine-focus sealed tube	1619 reflections with *I* > 2σ(*I*)
graphite	*R*_int_ = 0.049
Detector resolution: 13.6612 pixels mm^-1^	θ_max_ = 27.5°, θ_min_ = 3.1°
ω scans	*h* = −7→7
Absorption correction: multi-scan (*CrystalClear*; Rigaku, 2005)	*k* = −16→16
*T*_min_ = 0.921, *T*_max_ = 0.921	*l* = −17→17
9446 measured reflections	

### Refinement

**Table:**

Refinement on *F*^2^	Primary atom site location: structure-invariant direct methods
Least-squares matrix: full	Secondary atom site location: difference Fourier map
*R*[*F*^2^ > 2σ(*F*^2^)] = 0.062	Hydrogen site location: inferred from neighbouring sites
*wR*(*F*^2^) = 0.167	H-atom parameters constrained
*S* = 1.06	*w* = 1/[σ^2^(*F*_o_^2^) + (0.0688*P*)^2^ + 0.9865*P*] where *P* = (*F*_o_^2^ + 2*F*_c_^2^)/3
2108 reflections	(Δ/σ)_max_ < 0.001
127 parameters	Δρ_max_ = 0.65 e Å^−^^3^
7 restraints	Δρ_min_ = −0.90 e Å^−^^3^

### Special details

**Table:**

Geometry. All e.s.d.'s (except the e.s.d. in the dihedral angle between two l.s. planes) are estimated using the full covariance matrix. The cell e.s.d.'s are taken into account individually in the estimation of e.s.d.'s in distances, angles and torsion angles; correlations between e.s.d.'s in cell parameters are only used when they are defined by crystal symmetry. An approximate (isotropic) treatment of cell e.s.d.'s is used for estimating e.s.d.'s involving l.s. planes.
Refinement. Refinement of *F*^2^ against ALL reflections. The weighted *R*-factor *wR* and goodness of fit *S* are based on *F*^2^, conventional *R*-factors *R* are based on *F*, with *F* set to zero for negative *F*^2^. The threshold expression of *F*^2^ > σ(*F*^2^) is used only for calculating *R*-factors(gt) *etc*. and is not relevant to the choice of reflections for refinement. *R*-factors based on *F*^2^ are statistically about twice as large as those based on *F*, and *R*- factors based on ALL data will be even larger.

### Fractional atomic coordinates and isotropic or equivalent isotropic displacement parameters (Å^2^)

**Table:**

	*x*	*y*	*z*	*U*_iso_*/*U*_eq_	
Cl1	0.13491 (18)	0.20125 (8)	0.11005 (7)	0.0477 (3)	
O5	0.3901 (6)	0.6009 (3)	0.1267 (2)	0.0640 (9)	
C2	0.7259 (7)	0.3883 (3)	0.2591 (3)	0.0472 (9)	
H2A	0.8394	0.3735	0.2155	0.057*	
C6	0.5616 (7)	0.5419 (3)	0.1489 (3)	0.0442 (9)	
C3	0.5618 (7)	0.4719 (3)	0.2396 (3)	0.0395 (8)	
C4	0.3922 (7)	0.4908 (4)	0.3048 (3)	0.0495 (10)	
H4A	0.2785	0.5457	0.2926	0.059*	
C7	0.7761 (9)	0.5359 (4)	0.0917 (3)	0.0584 (11)	
H7A	0.7509	0.5840	0.0361	0.088*	
H7B	0.7913	0.4650	0.0676	0.088*	
H7C	0.9244	0.5550	0.1347	0.088*	
O4	0.0611 (7)	0.0968 (3)	0.0755 (3)	0.0790 (11)	
O3	−0.0390 (8)	0.2749 (3)	0.0608 (3)	0.0814 (11)	
O2	0.1393 (7)	0.2069 (3)	0.2155 (2)	0.0809 (12)	
C5	0.3935 (8)	0.4281 (4)	0.3873 (3)	0.0589 (12)	
H5A	0.2811	0.4403	0.4320	0.071*	
N1	0.5561 (8)	0.3493 (3)	0.4037 (3)	0.0607 (10)	
H1A	0.5546	0.3108	0.4562	0.073*	
O1	0.3763 (6)	0.2204 (3)	0.0851 (2)	0.0645 (6)	
C1	0.7196 (9)	0.3275 (4)	0.3430 (3)	0.0575 (11)	
H1B	0.8296	0.2716	0.3571	0.069*	

### Atomic displacement parameters (Å^2^)

**Table:**

	*U*^11^	*U*^22^	*U*^33^	*U*^12^	*U*^13^	*U*^23^
Cl1	0.0506 (6)	0.0518 (6)	0.0440 (5)	0.0087 (4)	0.0186 (4)	0.0039 (4)
O5	0.065 (2)	0.064 (2)	0.062 (2)	0.0170 (16)	0.0035 (15)	0.0069 (15)
C2	0.051 (2)	0.043 (2)	0.050 (2)	0.0003 (17)	0.0159 (18)	−0.0036 (17)
C6	0.049 (2)	0.042 (2)	0.041 (2)	−0.0013 (17)	0.0036 (17)	−0.0046 (16)
C3	0.0386 (18)	0.0403 (19)	0.0396 (18)	−0.0047 (15)	0.0058 (15)	−0.0044 (15)
C4	0.042 (2)	0.056 (2)	0.052 (2)	0.0015 (18)	0.0117 (17)	−0.0060 (19)
C7	0.066 (3)	0.067 (3)	0.045 (2)	−0.002 (2)	0.017 (2)	0.009 (2)
O4	0.085 (3)	0.060 (2)	0.097 (3)	−0.0072 (19)	0.032 (2)	−0.0063 (19)
O3	0.100 (3)	0.079 (2)	0.065 (2)	0.037 (2)	0.009 (2)	0.0155 (18)
O2	0.094 (3)	0.109 (3)	0.0421 (18)	0.030 (2)	0.0173 (17)	0.0069 (17)
C5	0.054 (3)	0.075 (3)	0.051 (2)	−0.014 (2)	0.020 (2)	−0.006 (2)
N1	0.073 (3)	0.058 (2)	0.052 (2)	−0.016 (2)	0.0125 (19)	0.0106 (18)
O1	0.0571 (11)	0.0787 (12)	0.0614 (11)	0.0010 (10)	0.0210 (10)	−0.0032 (10)
C1	0.067 (3)	0.046 (2)	0.060 (3)	0.000 (2)	0.011 (2)	0.007 (2)

### Geometric parameters (Å, °)

**Table:**

Cl1—O2	1.421 (3)	C4—C5	1.365 (6)
Cl1—O1	1.427 (3)	C4—H4A	0.9300
Cl1—O3	1.427 (3)	C7—H7A	0.9600
Cl1—O4	1.437 (4)	C7—H7B	0.9600
O5—C6	1.202 (5)	C7—H7C	0.9600
C2—C1	1.372 (6)	C5—N1	1.332 (6)
C2—C3	1.386 (5)	C5—H5A	0.9300
C2—H2A	0.9300	N1—C1	1.321 (6)
C6—C7	1.492 (6)	N1—H1A	0.8600
C6—C3	1.509 (5)	C1—H1B	0.9300
C3—C4	1.384 (5)		
			
O2—Cl1—O1	109.8 (2)	C3—C4—H4A	120.4
O2—Cl1—O3	110.7 (2)	C6—C7—H7A	109.5
O1—Cl1—O3	111.0 (2)	C6—C7—H7B	109.5
O2—Cl1—O4	109.6 (2)	H7A—C7—H7B	109.5
O1—Cl1—O4	107.8 (2)	C6—C7—H7C	109.5
O3—Cl1—O4	107.9 (2)	H7A—C7—H7C	109.5
C1—C2—C3	119.5 (4)	H7B—C7—H7C	109.5
C1—C2—H2A	120.2	N1—C5—C4	119.8 (4)
C3—C2—H2A	120.2	N1—C5—H5A	120.1
O5—C6—C7	123.0 (4)	C4—C5—H5A	120.1
O5—C6—C3	118.6 (4)	C1—N1—C5	123.1 (4)
C7—C6—C3	118.4 (3)	C1—N1—H1A	118.5
C4—C3—C2	118.9 (4)	C5—N1—H1A	118.5
C4—C3—C6	119.2 (3)	N1—C1—C2	119.4 (4)
C2—C3—C6	121.9 (3)	N1—C1—H1B	120.3
C5—C4—C3	119.3 (4)	C2—C1—H1B	120.3
C5—C4—H4A	120.4		
			
C1—C2—C3—C4	−1.1 (6)	C2—C3—C4—C5	1.1 (6)
C1—C2—C3—C6	179.5 (4)	C6—C3—C4—C5	−179.4 (4)
O5—C6—C3—C4	−12.0 (5)	C3—C4—C5—N1	−0.4 (6)
C7—C6—C3—C4	167.1 (4)	C4—C5—N1—C1	−0.3 (7)
O5—C6—C3—C2	167.4 (4)	C5—N1—C1—C2	0.3 (7)
C7—C6—C3—C2	−13.4 (5)	C3—C2—C1—N1	0.4 (6)

### Hydrogen-bond geometry (Å, °)

**Table:**

*D*—H···*A*	*D*—H	H···*A*	*D*···*A*	*D*—H···*A*
N1—H1A···O1^i^	0.86	2.14	2.896 (5)	146
C1—H1B···O5^ii^	0.93	2.49	2.963 (5)	112
C2—H2A···O3^iii^	0.93	2.59	3.435 (6)	151
C5—H5A···O4^i^	0.93	2.46	3.332 (6)	156
C7—H7B···O3^iii^	0.96	2.58	3.488 (6)	158

Symmetry codes: (i) *x*, −*y*+1/2, *z*+1/2; (ii) −*x*+1, *y*−1/2, −*z*+1/2; (iii) *x*+1, *y*, *z*.
